# Ongoing Peritoneal Dialysis Training at Home Allows for the Improvement of Patients’ Empowerment: A Single Center Experience

**DOI:** 10.3390/jcm13020411

**Published:** 2024-01-11

**Authors:** Sabrina Milan Manani, Grazia Maria Virzì, Niccolò Morisi, Davide Marturano, Ilaria Tantillo, Anna Giuliani, Nunzia Miranda, Alessandra Brocca, Gaetano Alfano, Gabriele Donati, Claudio Ronco, Monica Zanella

**Affiliations:** 1 Department of Nephrology, Dialysis and Transplant, San Bortolo Hospital, 36100 Vicenza, Italygraziamaria.virzi@gmail.com (G.M.V.); davide.marturano@aulss8.veneto.it (D.M.); nunzia.miranda@aulss8.veneto.it (N.M.);; 2 IRRIV—International Renal Research Institute Foundation, 36100 Vicenza, Italy; 3 Nephrology Dialysis and Renal Transplantation Unit, University of Modena and Reggio Emilia, 41121 Modena, Italy; gaetano.alfano@unimore.it (G.A.); gabriele.donati@unimore.it (G.D.); 4 Department of Clinical Chemistry and Hematology Laboratory, San Bortolo Hospital, Viale F Rodolfi, 37, 36100 Vicenza, Italy

**Keywords:** peritoneal dialysis, ongoing training at home, empowerment, outcomes

## Abstract

Introduction: Peritoneal dialysis (PD), as a home treatment, ensures better patient autonomy and lower intrusiveness compared to hemodialysis. However, choosing PD comes with an increased burden of responsibility that the patient may not always be able to bear, due to advanced age and deteriorating health condition. Various approaches have been explored to address this issue and mitigate its primary complications. In this study, we aim to present the ongoing PD training at-home program implemented by the Vicenza PD Center, and evaluate its impact on patients’ prognoses. Material and Methods: We enrolled 210 patients who underwent PD at Vicenza Hospital between 1 January 2019 and 1 January 2022 for a minimum of 90 days. Each patient was observed retrospectively for one year. We categorized the patients into three groups based on their level of autonomy regarding their PD management: completely independent patients; patients able to perform some parts of the PD method on their own, while the remaining aspects were carried out by a caregiver; and patients who required complete assistance from a caregiver, like in the assisted PD program (asPD). Results: A total of 70% of the PD population were autonomous regarding their PD therapy, 14% had an intermediate degree of autonomy, and 16% were entirely dependent on caregivers. The PD nurses performed a median of four home visits per patient per year, with a tendency to make more visits to patients with a lower degree of autonomy. All the groups achieved similar clinical outcomes. At the end of the year of observation, only 6% of the patients witnessed a decline in their autonomy level, whereas 7% demonstrated an enhancement in their level of autonomy, and 87% remained stable. Conclusions: A home care assistance program ensures clinical support to a household with the purpose of improving the empowerment of the PD population and reducing the prevalence of assisted PD. Ongoing PD training at home helps patients to maintain a stable degree of autonomy and stay in their home setting, even though they present with relative attitudinal or social barriers.

## 1. Introduction

The constant ageing of the global population is presenting new challenges in the healthcare sector, particularly with regard to the growing epidemic of chronic kidney failure (CKD) and its associated complications [1,2,3]. As the number of CKD patients continues to rise, there will be a corresponding increase in end-stage kidney disease (ESKD) patients who require renal replacement therapy. Not all these patients will have the opportunity to undergo a kidney transplant, necessitating a decision regarding the choice of dialysis modality: hemodialysis (HD) or peritoneal dialysis (PD). The guidelines recommend involving the patient and their relatives in the decision-making process, while healthcare professionals analyze the various medical, social, and economic factors [4,5]. Although no clear superiority of one method over the other has been established, PD offers several advantages. This method employs a more physiological purification system and helps to preserve residual kidney function for a longer period. Moreover, it allows patients to maintain a good quality of life, as it can be performed at home and is modular [6]. However, choosing PD comes with an increased burden of responsibility that the patient may not always be able to bear, due to advanced age and deteriorating health condition [7]. Consequently, the patient often relies on the assistance of a caregiver [8]. Typically, this caregiver is a family member, friend, or occasionally a hired individual who assists the patient with their daily routine and treatments. Over time, this situation can lead to a decline in quality of life. Various approaches have been explored to address this issue and mitigate its primary complications [9]. The Vicenza Dialysis Center, in particular, has implemented a program of nursing visits to the patient’s home, described in the next section. In this study, we aim to present the ongoing PD training program of the PD Center of Vicenza and evaluate its impact on patients’ prognoses, compliance, and complications. In particular, we evaluated the PD adequacy, peritonitis, exit-site infection rates, and days of hospitalization among the patients with varying degrees of autonomy at the time of enrollment.

## 2. Methods

### 2.1. Ongoing PD Training at Home at the Vicenza PD Center

**Figure 1:**
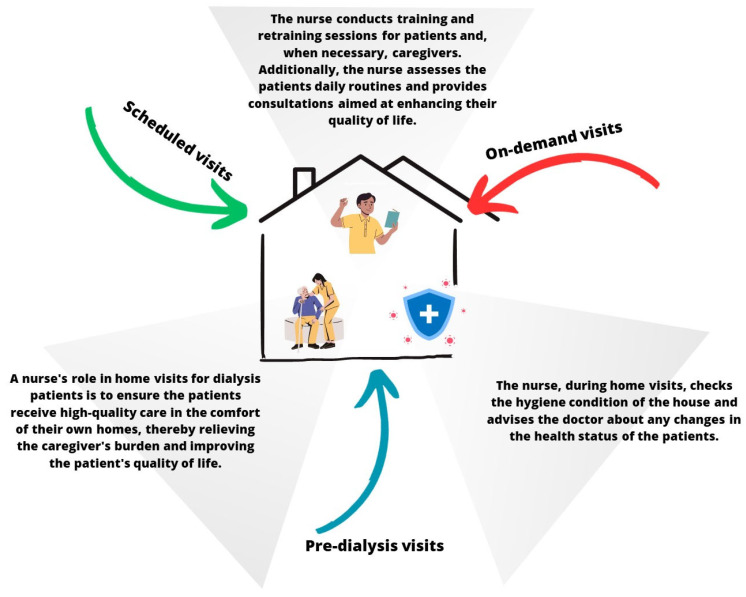
Visualization of the ongoing training by home healthcare nurses for peritoneal dialysis.

At the PD Center at San Bortolo Hospital in Vicenza, the nursing team adheres to the regulations set forth by the Italian government and follows the guidelines established by the Italian Society of Nephrology [10]. Within the team, two nurses are specifically designated for patient and caregiver training, as well as for home monitoring. For newly enrolled peritoneal dialysis patients, the team assesses their home to ensure suitability before commencing the treatment (duration time = 2 h). The training phase is then divided into two parts: an initial session at the hospital (duration = 3 h) followed by a subsequent session at the patient’s home (duration = 3/4 h for 2 days). After the training period, the team continues to support the patient by conducting home visits every three months (a routine home visit duration is similar to an in-hospital visit), which can be adjusted based on the patient’s needs. During a home visit, nurses record clinical parameters, such as weight, blood pressure, and bioimpedance analysis, check the medical and dialytic therapy, and verify patient compliance. These visits serve as an opportunity for the patient and caregiver to review the procedures learned during training, while also reinforcing guidelines for maintaining a healthy lifestyle (Figure 1).

### 2.2. Study Design

This was a one-year retrospective observational study conducted at the PD Center at San Bortolo Hospital in Vicenza, Italy (one year is the observational period for each patient).

All patients aged 18 and above, who initiated PD on or after 1 January 2019 until 1 January 2022 (patient enrollment period: 3 years), and had a minimum treatment duration of 90 days, were considered eligible for this study. All patients were provided with information about the experimental protocol and this study’s objectives. Patients who declined to provide consent were also excluded from this study. This study adhered to the principles outlined in the Declaration of Helsinki, and the research protocol and informed consent were approved by the ethics committee of San Bortolo Hospital (approval number 44/23, date 26 May 2023).

Demographic and clinical characteristics, along with the frequency of visits to nursing homes, were gathered from the MEDWARE^®^ database (Sined s.r.l., Bologna, Italy). Body weight and height measurements were utilized to calculate the body mass index (BMI) for each participant. Patient comorbidities were assessed using the age-adjusted Charlson comorbidity index (ICC) [11].

The effectiveness of PD was evaluated by examining the total (dialytic + kidney) weekly urea clearance normalized by the volume of distribution (wKT/V), and the total (dialytic + kidney) weekly creatinine clearance (wCCr) (adjustment for BSA) [12].

All parameters were recorded at the end of the observational period of one year.

The incidence of peritonitis and exit-site infections was expressed as the number of episodes per patient per year [13,14].

Blood and peritoneal fluid samples were collected during scheduled and urgent visits in accordance with clinical practices. Laboratory data and parameters related to PD were obtained at the onset of the observation period.

### 2.3. Definition of Patient Groups

We categorized the patients into three groups based on their level of autonomy and the degree of assistance provided by the caregiver:

Group A: Patients capable of independently performing all aspects of the PD method, including evaluating the exit site and managing the dialysis fluid, regardless of the presence of a caregiver.

Group B: Patients who are able to perform some phases of the PD method on their own, while the remaining aspects are carried out by the caregiver.

Group C: Patients who require complete assistance from the caregiver for all stages of the PD method, like in an assisted PD program [15].

### 2.4. Endpoints

The primary objective of this study is to assess the outcomes for patients undergoing PD, such as PD adequacy and key complications (peritonitis and exit-site infections).

We aim to compare these outcomes among patients with varying degrees of autonomy at the time of enrollment.

As the secondary objective, we will describe the autonomy degree at the beginning and at the end of the observational period.

### 2.5. Statistical Analysis

Statistical analyses were performed using SPSS statistics 26.0 software (IBM Corp., Armonk, NY, USA). Normality tests were performed using the Kolmogorov–Smirnov test. It was decided to consider a value of *p* < 0.05 as statistically significant. Categorical variables were expressed as percentages; we performed chi-squared and Fisher’s exact tests to discover the associations between categorical variables. Continuous variables were expressed with the median and interquartile range; Kruskal–Wallis H tests and post hoc pairwise Dunn tests were performed to compare the median value among the groups.

## 3. Results

### 3.1. Population Characteristics

A total of 214 patients met the inclusion criteria for this study. Four patients were excluded because they declined to provide consent. Finally, we enrolled 210 patients; 67% were male (n = 140). The distribution of the population data deviated from normality. The median age of the participants at study entry was 62 years (interquartile range (IQR) 50; 73 years). The median age-adjusted Charlson comorbidity index (CCI) was 5 (IQR 4; 7). Automated PD (APD) was used for 79% (n = 166) of the studied population and continuous ambulatory PD (CAPD) for 21% (n = 44) of the cases. Just 41 patients using APD performed continuous cyclic PD (CCPD), while most of the APD patients performed night intermittent PD (NIPD). The population median wKT/V was 1.89 (IQR 1.57; 2.12), while the wCCr was 49.41 mL/min (IQR 32.9; 72.6). The clinical data for this study’s total population are reported in Table 1.

Among the enrolled subjects, 70% (n = 146) were classified as completely independent (Group A), 14% (n = 29) had an intermediate degree of autonomy (Group B), while 16% (n = 35) were entirely dependent on caregivers for the methodology.

Regardless of the patient’s autonomy status, caregivers were identified in 66% of the cases (n = 139). All 44% of our PD population arepatients without a caregiver (Group A).

Only 10% of the caregivers were salaried individuals (n = 13), while the majority were relatives or friends of the patients.

The main clinical and laboratory characteristics, divided by groups, are summarized in Table 2. A statistically significant difference was observed for the investigated parameter of age-adjusted CCI (*p* = 8 × 10^−5^) among the three groups, with the scores increasing as the degree of autonomy decreased. Moreover, there was a tendency to decline the use of APD and to use CAPD for patients with less autonomy.

**Figure 2:**
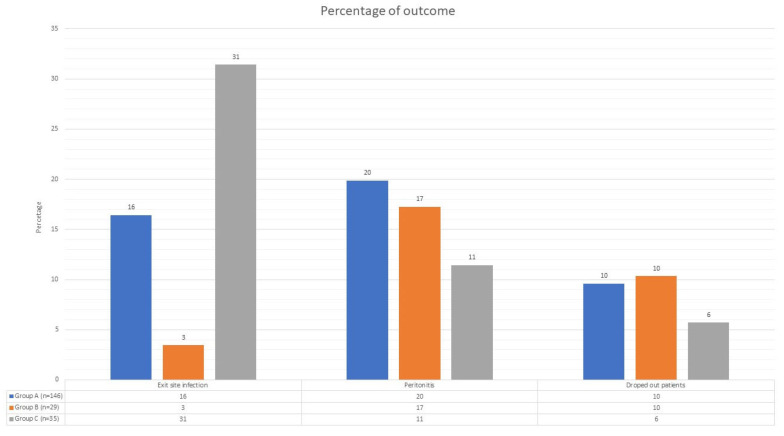
Clinical outcomes. This figure consists of three groups, each containing three histograms. Each group represents an adverse event during peritoneal dialysis (exit-site infections, peritonitis, or dropout), and each histogram signifies a different level of patient autonomy (blue for fully autonomous patients, orange for partially autonomous patients, and gray for non-autonomous patients). The *y*-axis represents the percentage. In the table beneath each group of histograms, you can see the number of each event divided by each level of patient autonomy.

The results for the primary objectives are summarized in Table 3 and Figure 2.

### 3.2. Drop Out

Initially, a total of 210 subjects were selected for this study; however, only 191 patients completed the follow-up. The primary reasons for withdrawal from the follow-up were patient death (n = 9), kidney transplantation (n = 6), and switching to HD (n = 4). Among the patients who did not complete the follow-up, only two shifted to HD due to sterile peritonitis, while the others did not experience peritoneal complications but needed to switch modality because of major abdominal surgery.

### 3.3. Home Visits and Hospitalizations

The median number of home visits is equivalent to four visits per patient per year, with an unequal distribution among the three groups (*p* = 0.001), with a tendency to make more visits to the patients with a lower degree of autonomy (see Table 2). In two cases, the nurses also performed training for patients admitted to a residential home.

Regarding the number of days of hospitalization, the median is 21 days (IQR 7; 44) per year with no statistically significant difference between the three groups (*p* = 0.81). Patients were admitted for acute illness and for rehabilitation, and during both they performed PD as scheduled.

### 3.4. Exit-Site Infections

Exit-site infections were observed at a frequency of 0.171 episodes per patient per year (n = 36). A Fisher’s test confirmed the absence of statistically significant differences between the three groups of patients (*p* = 0.136). Gram-positive (Gram+) bacteria were isolated in 47% (n = 17) of the cases, while the rest were Gram-negative (Gram−) or contaminants. Antibiotic therapy was topical in all the cases, with the addition of a systemic drug in 44% of the cases (n = 15). A clinical resolution was achieved for all the cases, requiring hospitalization in 11% (n = 4) of the cases due to the need for intravenous therapy.

### 3.5. Peritonitis

During this study, the frequency of peritonitis was 0.18 episodes per patient-year (n = 38). Using Fisher’s test, no statistically significant differences were identified between the three groups of patients (*p* = 0.44). For the peritoneal fluid culture before therapy with antibiotics, 29% (n = 10) had sterile fluid, 47% (n = 16) were Gram−, and 23% (n = 8) were Gram+. All the episodes were resolved with antibiotic therapy with a hospitalization rate of 16% (n = 6).

### 3.6. Autonomy Degree

As previously described, 191 patients completed this one-year study. Among these, 11 (6%) patients witnessed a decline in their autonomy level, whereas 14 (7%) patients demonstrated an enhancement in their level of autonomy, and 166 (87%) patients remained stable.

## 4. Discussion

The present study described the Vicenza PD Center experience regarding the follow-up for PD patients. The results indicate that there are no significant differences among the three examined groups when analyzing the common complications of peritoneal dialysis, despite a higher number of home visits to the group with lower autonomy.

In our clinical practice, the in-person visits are made by physicians and nurses and, moreover, home visits are performed by nurses both routinely and as needed.

Patients with decreased autonomy may require additional care and assistance in performing PD exchanges. They rely on caregivers or healthcare professionals to help them with the procedure, including setting up the equipment, monitoring the process, and managing potential complications, until they have the full support provided by the assisted PD modality (asPD) [15].

In addition to home visits, the patients hospitalized for a limited period, either in acute care units or rehabilitation centers, continued PD thanks to the training provided by the PD team to the nurses, avoiding a temporary shift to HD. In the same way, the patients in residential homes were treated with CAPD, because of the presence of health personnel during the day.

Similarly, we scheduled CAPD exchanges during the patients’ daily activities for those in Group C who required support for many aspects of their lives to reduce the impact of nighttime caregiving duties. Furthermore, none of the patients were permanently bedridden, and CAPD allowed them to maintain their daily routines unchanged.

The enrollment characteristics were similar among the three groups, but with a statistical difference observed in terms of age and the Charlson comorbidity index (CCI). Specifically, the patients in Group C who required complete assistance were older and had higher CCI scores.

Furthermore, while both the PTH (parathyroid hormone) and phosphate levels were higher for Group A, only the PTH exhibited a statistical significance. This difference may be attributed to decreased adherence to the prescribed diet in the case of reduced caregiver oversight [18,19].

As previously described in the literature, we observed that age and a high ICC were more commonly associated with a lower grade of autonomy [20]. Typically, these factors are also linked to worse outcomes and an increased occurrence of peritoneal complications. Notably, peritonitis stands out as the leading cause of mortality among elderly PD patients [21]. However, we discovered that the incidence of peritonitis was similar across our three patient groups. This implies that, in our experience, peritonitis occurs independently of a patient’s autonomy status. Based on our results, we observed that assisted PD patients do not present with a higher rate of complications, such as exit-site infections and peritonitis, compared to other groups. In our opinion, a home-based ongoing-training PD program could help to improve compliance for PD patients who require assistance from a caregiver for all stages of the PD method. Just two patients dropped out because of peritonitis and both came from Group A. Similar findings were observed regarding dialysis adequacy and other complications under study, such as exit-site infections and hospitalizations, without statistical differences among three groups. The repeated and reinforced information given during the ongoing PD training could have contributed to enabling patients with reduced or no autonomy to achieve satisfactory performance results. To achieve this result, we performed more on-demand visits to patients from Group C.

It has been already demonstrated that, in chronic conditions, a collaborative process between patients and professionals and the consequent empowerment of patients are central to improving care and outcomes [22,23,24,25]. Based on our experience, our home-based ongoing-training PD program improves the patient’s compliance and relationship with the care team, increasing collaboration with and confidence in the nurses and specialists. In our opinion, this type of program could have an important impact on clinical practice, with a strong benefit to the patient’s compliance and outcomes. Furthermore, during this program, collaboration with the care team grew and relationships with the nurses and doctors became better. We think that this ongoing-training PD program could be used to implement the use of PD in the ESKD population, offering this dialytic approach also to patients with reduced autonomy.

In the literature, the percentage of the distribution between self-sufficient patients and caregiver-supported or nurse/family-assisted PD is variable around the world, but is growing because of the aging of the population. In a cohort of a French-language PD registry, almost 50% of the incident patients were assisted with their PD treatment [26]. A similar result is described in a recent paper about Thai PD patients, where assisted PD was required by 57% of the population [27].

In our experience, the percentage of non-self-sufficiency s 16%, while 44% of the total PD population was able to perform PD completely alone.

We speculate that the ongoing PD training at home allows for the encouragement of a proactive attitude among the patients, while reducing the percentage of asPD in comparison to the literature.

A total of 86.9% of our patients remained stable and 7.3% improved their autonomy level, while only 5.7% needed a higher level of support, and none shifted to HD for a lack of autonomy or caregivers.

Our care program is supported by previous literature data. Oliver et al. suggested a mixed model where home visits ensure the correct level of care according to the patient’s needs and complexity: in this way, 23% of asPD patients gradually improved to self-PD [20,28].

Our study has limitations. First of all, it is worth noting that these conclusions are based on experiences at the Vicenza center. We performed this study to propose an alternative model that could be adopted by other peritoneal units. After this happens, it would be useful to repeat the study with a multicenter approach. Secondly, we did not investigate the socioeconomic state of our patients [29], the public health aspects, or the nurses’ and doctors’ points of view. This pilot study can be considered hypothesis generating, and stimulate further explorations: additional research and studies of different settings and populations would be beneficial to validate these findings.

## 5. Conclusions

PD can be a viable treatment option for patients with ESKD, even in cases of reduced autonomy, as long as they have a caregiver. A collaborative effort involving the patient, caregiver, and healthcare operator is crucial for the success of PD [30,31,32].

The aim of the Vicenza ongoing PD training at home is a patient-centered educational approach, which can ensure that the necessary care is tailored to the different levels of autonomy, empower patients to their fullest potential, and is effective and safe.

Based on our pilot experience, we think that this ongoing PD training at home is a good approach, and we want to keep working in this way. Our preliminary results suggest that such a patient-centered educational approach is effective for providing the necessary care tailored to the different levels of autonomy, while emphasizing the importance of continuous support and training for PD patients at home. In this context, it is essential to continue monitoring and studying these patients to validate the findings and to thoroughly assess the long-term outcomes.

## Figures and Tables

**Table 1:** Clinical characteristics of total population and guidelines references.

	Patients’ Characteristics		Total Population (n = 210)	Guidelines (Reference)
Clinical data	Age; median (IQR)		62 (50; 73)	
		Gender, male; n (%)	21 (10)	
		>80 years old; n (%)	140 (67)	
	CCI; median (IQR)		5 (4; 7)	
	BMI; median (IQR)		26.03 (22.78; 29.53)	
Laboratory	Hemoglobin, g/L; median (IQR)		116.37 (110.18; 122.81)	95–115 [16]
	Phosphate, mg/dL; median (IQR)		4.72 (4.10; 5.84)	2.5–4.5 [16]
	Albumin, g/dL; median (IQR)		3.89 (3.63; 4.10)	>3.5 [17]
	PTH, pg/dL; median (IQR)		123.50 (60.50; 223)	2–9 times the upper normal limit [16]
Peritoneal data	APD, n (%)		166 (79)	
		CCPD; n (%)	125 (60)	
		NIPD; n (%)	41 (19)	
	CAPD, n (%)		44 (21)	
	Total wKT/Vurea; median (IQR)		1.89 (1.57; 2.12)	1.7–2.0 [12]
	Total wCCr, mL/min; median (IQR)		49.41 (33.06; 72.42)	45 L/1.73 m^2^ BSA [12]

Legend: Interquartile range (IQR); Charlson comorbidity index (CCI); body mass index (BMI); number (n); percentages (%); automated peritoneal dialysis (APD); continuous cycling peritoneal dialysis (CCPD); nocturnal intermitted peritoneal dialysis (NIPD); continuous ambulatory peritoneal dialysis (CAPD); total weekly KT/V urea (Total wKT/Vurea); total weekly creatinine clearance (Total wCCr).

**Table 2:** Clinical characteristics of three groups.

	Patients’ Characteristics	Group A (n = 146)	Group B (n = 29)	Group C (n = 35)	*p* Value
Clinical data	Age; median (IQR)	57 (47; 71.25)	68 (62.50; 74)	70 (61; 81)	15 × 10^;5^
	>80 years old; n (%)	10 (7)	2 (7)	9 (26)	
	Gender, male; n (%)	99 (68)	22 (76)	19 (54)	0.62
	CCI; median (IQR)	5 (3; 8)	7 (5; 7)	8 (6; 8)	8 × 10^−5^
	BMI; median (IQR)	26.03 (22.76; 28.43)	28.44 (23.27; 30.97)	24.12 (21.6; 27.25)	0.07
Laboratory	Hemoglobin, g/L; median (IQR)	116 (109; 122.75)	118 (111.75; 125)	118 (110.25; 122.43)	0.67
	Phosphate, mg/dL; median (IQR)	4.80 (4.13; 5.98)	4.67 (3.92; 5.42)	4.52 (4.13; 5.41)	0.29
	Albumin, g/dL; median (IQR)	3.90 (3.67; 4.10)	4 (3.70; 4.32)	3.66 (3.49; 3.90)	0.01
	PTH, pg/dL; median (IQR)	131.67 (69.62; 252.25)	66.33 (36; 180.81)	97.37 (50; 187.75)	0.01
Peritoneal data	APD, n (%)	127 (87)	19 (66)	20 (57)	1 × 10^−4^
	NICP, n (%)	101 (69)	13 (45)	11 (31)	
	CCPD, n (%)	26 (18)	6 (21)	9 (26)	
	CAPD, n (%)	19 (13)	10 (34)	15 (43)	
	Total wKT/Vurea; median (IQR)	1.84 (1.50; 2.15)	1.94 (1.47; 2.21)	1.94 (1.47; 2.21)	0.88
	Total wCCr, mL/min; median (IQR)	47.51 (32.68; 71.70)	49.72 (30.76; 73.60)	52.46 (41.46; 84.52)	0.56

Legend: Interquartile range (IQR); Charlson comorbidity index (CCI); body mass index (BMI); number (n); percentages (%); automated peritoneal dialysis (APD); continuous cycling peritoneal dialysis (CCPD); nocturnal intermitted peritoneal dialysis (NIPD); continuous ambulatory peritoneal dialysis (CAPD); total weekly KT/V urea (Total wKT/Vurea); total weekly creatinine clearance (Total wCCr).

**Table 3:** Clinical outcomes.

	Total	Group A (n = 146)	Group B (n = 29)	Group C (n = 35)	*p* Value
Number of home visits per patient/year (IQR)	4 (1; 7)	3 (1; 5)	4 (2.5; 7)	7 (3.5; 11)	0.001
Exit-site infection, n (APR)	36 (0.171)	24 (0.164)	1 (0.034)	11 (0.314)	0.14
Peritonitis, n (APR)	38 (0.180)	29 (0.21)	5 (0.172)	4 (0.114)	0.44
Dropped out patients, n (APR)	19 (0.090)	14 (0.096)	3 (0.103)	2 (0.057)	0.81
Days of hospitalization; n (IQR)	21 (7; 44)	21 (7; 43.50)	16 (6; 16)	26 (9.50; 26)	0.82

Legend: Interquartile range (IQR); number (n); annualized patient rate (APR) refers to total patients or the number of patients in a single group.

## Data Availability

The data presented in this study are available on request from the corresponding author. The data are not publicly available due to our internal policy.
